# Phosphorylation Targets of DNA-PK and Their Role in HIV-1 Replication

**DOI:** 10.3390/cells9081907

**Published:** 2020-08-16

**Authors:** Andrey Anisenko, Marina Kan, Olga Shadrina, Anna Brattseva, Marina Gottikh

**Affiliations:** 1 Chemistry Department and Belozersky Institute of Physico-Chemical Biology, Lomonosov Moscow State University, Moscow 119234, Russia; oashadrina92@gmail.com (O.S.); gottikh@belozersky.msu.ru (M.G.); 2 Faculty of Bioengineering and Bioinformatics, Lomonosov Moscow State University, Moscow 119234, Russia;; marinakan2205@mail.ru (M.K.); anabellabr@mail.ru (A.B.)

**Keywords:** DNA-PK, HIV-1, DNA-damage, HIV-1 transcription regulation, post-integrational repair

## Abstract

The DNA dependent protein kinase (DNA-PK) is a trimeric nuclear complex consisting of a large protein kinase and the Ku heterodimer. The kinase activity of DNA-PK is required for efficient repair of DNA double-strand breaks (DSB) by non-homologous end joining (NHEJ). We also showed that the kinase activity of DNA-PK is essential for post-integrational DNA repair in the case of HIV-1 infection. Besides, DNA-PK is known to participate in such cellular processes as protection of mammalian telomeres, transcription, and some others where the need for its phosphorylating activity is not clearly elucidated. We carried out a systematic search and analysis of DNA-PK targets described in the literature and identified 67 unique DNA-PK targets phosphorylated in response to various in vitro and/or in vivo stimuli. A functional enrichment analysis of DNA-PK targets and determination of protein–protein associations among them were performed. For 27 proteins from these 67 DNA-PK targets, their participation in the HIV-1 life cycle was demonstrated. This information may be useful for studying the functioning of DNA-PK in various cellular processes, as well as in various stages of HIV-1 replication.

## 1. Introduction

DNA-dependent protein kinase (DNA-PK) is a heterotrimeric complex that consists of Ku70 (XRCC6), Ku80 (XRCC5), and DNA-PKcs (PRKDC). The last one belongs to the phosphatidyl inositol 3-kinase-like kinases (PIKKs) family [1]. DNA-PK is a major component of DNA double-strand break (DSB) repair system, which initiates the non-homologous end joining pathway (NHEJ). DNA-PK also participates in V(D)J and class-switch recombination [1]. More and more non-canonical functions of DNA-PK have recently been reported [2]; for instance, this complex has been shown to participate in ageing processes and metabolism regulation [3], transcription [4], and telomere maintenance [5,6].

NHEJ pathway begins with the recognition of a DNA DSB (double-strand break) by Ku70/Ku80 heterodimer. After that, DNA-PKcs is recruited to the DNA damage site and binds to the Ku70/Ku80-DNA complex, which triggers its kinase activity. The phosphorylating activity of this enzyme is crucial for efficient recruitment and activity regulation of DNA repair factors [1]. Activation of DNA-PK due to binding to DNA is a canonical activation mode, which is typical for such cellular functions of DNA-PK as DSB repair, V(D)J and class-switch recombination, and foreign DNA sensing [1,7,8]. 

However, it has recently been shown that DNA-PK activation is also possible under its interaction with RNA. This non-canonical activation has been described for RNA helicase A and heterogeneous nuclear ribonucleoprotein A1 phosphorylation [5,6,9]. 

**Figure 1:**
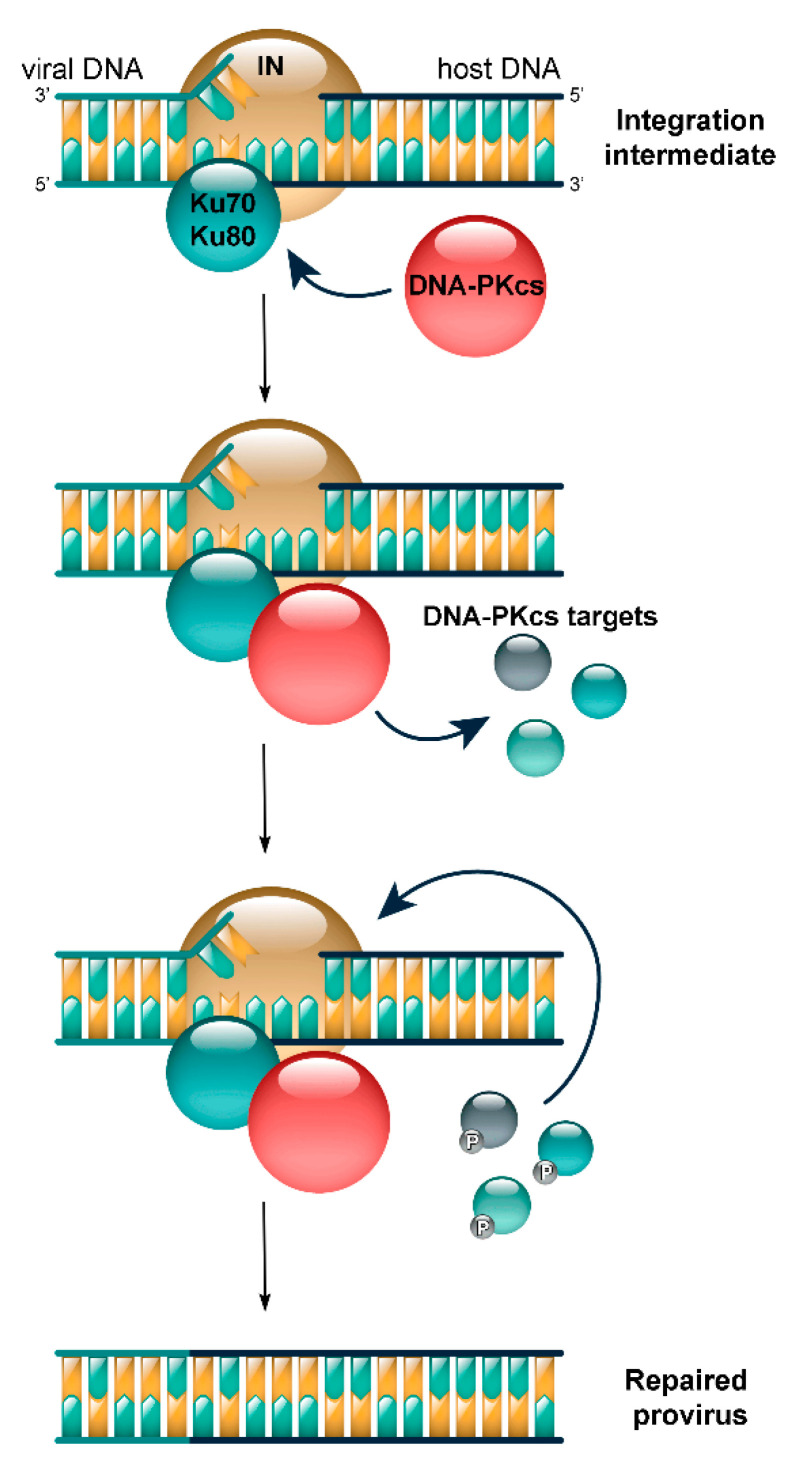
Model of HIV-1 post-integrational DNA repair based on our previously published data [10]. HIV-1 integrase (IN) that marks integration sites recruits the Ku70/Ku80 heterodimer by direct interaction with the Ku70 subunit; then, the catalytic subunit of DNA-PK (DNA-PKcs) binds to this complex and phosphorylates some unknown protein targets that results in DNA repair. This process strongly depends on the interaction between IN and Ku70.

One more example of the non-canonical activation of DNA-PKcs has been discovered by studying how the DNA-PK complex is involved in HIV-1 post-integrational repair (PIR) [10]. The integration of HIV-1 genome is vital for efficient production of new viral particles. However, this process is the key source of danger for further viral reproduction, because HIV-1 DNA integration into the cellular DNA causes its damage [11]. The integration of viral DNA results in the formation of proviral DNA with five-nucleotide single-strand gaps in genomic DNA on both sides of the viral DNA, and the unmatched dinucleotides located on the 5′ ends of the viral DNA (the so-called integration intermediate) (Figure 1). Cellular systems of DNA repair restore the integrity of this integration intermediate, which eventually results in the formation of a provirus—a template for the production of new virions [11]. 

Using a modified variant of gag-alu specific PCR [12], we found that DNA-PK components, Ku70, Ku80, and DNA-PKs, are involved in PIR of the integration intermediate [10]. Moreover, the recruitment of DNA-PK to viral DNA integration sites and the activation of its catalytic subunit is mediated by the direct interaction of HIV-1 integrase (IN) (the enzyme accomplishing the integration of viral DNA into the cell’s genome) and Ku70, a part of DNA-PK [10] (Figure 1). The impaired interaction between these proteins decreases the infectivity of VSV-G-pseudotyped HIV-like particles. We also showed that the phosphorylating activity of DNA-PKcs is crucial for the efficient accomplishment of PIR, since the low-molecular specific inhibitor of DNA-PKcs kinase activity Nu7441 decreases both the PIR efficiency and the pseudovirus infectivity [10]. Thus, it is obvious, that DNA-PK as well as its targets, phosphorylated during PIR, are necessary for the efficient completion of this process. Importantly, there are no double-stranded DNA breaks in the product of viral DNA integration. In this case, DNA-PK activation is triggered by IN binding to Ku70, since IN mutations disturbing this binding significantly reduce the PIR efficiency and disrupt the pseudovirus sensitivity to Nu7441 [10]. Therefore, this is an example of non-canonical activation of DNA-PK due to protein–protein binding.

The necessity of DNA-PK phosphorylating activity has also been shown for some other cellular processes. For example, phosphorylation of TRIM28 by DNA-PK converts it to an elongation factor during transcription regulation [13]. Just recently, the necessity of RNA polymerase II phosphorylation by DNA-PK for the efficient transcription from the HIV-1 promoter and reactivation of the latent provirus has been clearly demonstrated [14]. However, the mechanism of DNA-PK activation during transcription remains obscure, since the DNA-binding component of DNA-PK, the Ku protein, has high affinity to DNA ends, whereas its ability to interact with internal DNA sequences is not proved clearly [15]. 

To date, there is a lack of systematic research of phosphoproteome alterations upon DNA-PK activation. In one of the most grand-scale studies aimed at finding DNA-PKcs targets, 26 proteins have been found to be extensively phosphorylated upon the activation of this kinase [16]. However, among these proteins, there are no some classical DNA-PK targets (e.g., p53), which indicates that this list is not complete. The lack of such systematic studies, unfortunately, complicates the understanding of details of regulation of DNA-PK-dependent processes including the further study of HIV-1 PIR and transcription regulation. 

In the present study, we first performed a search and analysis of DNA-PK targets described in literature. We managed to collect information on 67 unique DNA-PK targets, phosphorylated in response to various stimuli in vitro and/or in vivo. The represented targets could be conventionally divided into the following functional groups: DNA-repair, cell response to heat, post-translational RNA processing, transcription regulation, and a less functionally homogeneous cluster with proteins involved in cell cycle regulation (e.g., TP53 and MDM2), RNA biogenesis regulation (e.g., Jun, POLR2A, and POU2F1), and response to exo- and endogenous stimuli (e.g., AKT1 and AKT2). We also separately analyzed the literature data on the involvement of the indicated DNA-PK targets in HIV-1 replication.

## 2. Cellular Functions of DNA-PK 

DNA-PK is a huge heterotrimeric complex comprised of the Ku70 and Ku80 that form the Ku-heterodimer and a 470-kDa catalytic subunit DNA-PKcs. The involvement of the DNA-PK protein complex, as well as its DNA-binding component Ku, in various cellular processes is being studied quite extensively [1,2,3,4,5,6]. Nevertheless, the functional role of DNA-PK has been thoroughly described only for DNA double-strand break repair by NHEJ pathway [1]. 

DNA-PKcs as a member of PIKKs family, together with two other kinases from the same family called ATM and ATR, maintains genome stability through regulation of the cellular DNA damage response (DDR). These kinases are activated in response to DNA damage, which leads to cell cycle arrest and DNA repair due to phosphorylation of different protein targets [1]. When activated, DNA-PKcs preferentially phosphorylates protein targets at canonical for PIKK S/T-Q sites, but the phosphorylation within non-S/T-Q contexts has also been shown [1].

DNA-PK mediates DSB repair by NHEJ-pathway, which is a major cell cycle independent repair pathway for this type of DNA lesions. The less error-prone way to repair DSB is a homologous recombination (HR), which is orchestrated by ATM. It takes place only in late S-G2 phases of cell cycle, and here NHEJ competes with HR pathway. Despite the significant difference between HR and NHEJ mechanisms in the DNA integrity restoration, DNA-PKcs may also participate in negative regulation of HR [17]. This regulation depends on kinase activity of DNA-PKcs [18], and may be explained by phosphorylation of ATM [19]. 

In the NHEJ pathway several principal stages can be distinguished: (1) DSB sensing; (2) recruitment of repair factors to the damage site and synapsis of DNA ends; (3) processing of DNA ends; and (4) ligation of these ends together. Ring-shaped Ku-heterodimer rapidly binds DNA ends after DSB formation [20,21] and increases the affinity of DNA-PKcs to DNA ends [22], which results in DNA-PK complex assembly and the activation of the catalytic subunit [23]. DNA-PK complex acts both as a scaffold platform for NHEJ participants (XLF, XRCC4, APLF, Ligase IV, etc.) that are essential for synapsis formation, end-processing, and ligation and as a kinase that modifies chromatin around the DSB, regulates the activity of repair factors as well as promotes DNA-PK disassembly from DSB sites to allow DNA ends’ ligation [1,23,24,25]. Noteworthy, DNA-PKcs autophosphorylation is important for DNA-repair regulation. DNA-PKcs undergoes autophosphorylation in a DNA-damage dependent manner at multiple S/T-Q (S2056, T2609, S2612, S2620, T2638, T2647, and T3950) as well as non-S/T-Q site (S2624) in vivo [26]. Neither S2056 nor T2609 is required for DNA-PKcs kinase activity, but both are important for DNA repair. The current model suggests that their phosphorylation causes conformational changes that promote DNA-PK disassembly from DSB sites to allow DNA-end ligation [1]. Another phosphorylation site with a known effect on DNA repair is located in the kinase domain (T3950) and may act to switch off DNA-PKcs kinase activity when phosphorylated [1]. 

V(D)J and class switch recombination during B- and T-cell differentiation is another example of a cellular process involving DNA-PK. The DSBs are generated in a programmed enzymatic manner in both processes, and as a result, the NHEJ pathway is an integral part of both processes. The absence or mutation of NHEJ factors results in defective recombination leading to immune deficiencies and/or predisposition to cancers such as leukemias and lymphomas [8,27]. 

Recently, DNA-PK has been identified as a foreign DNA sensor in the cytoplasm that activates innate immunity. The DNA binding properties of Ku are obligatory for this activity [7,28]. For some viruses such as Vaccinia virus, Human adenovirus 5, and Herpes simplex virus 1, the mechanisms that counteract the DNA sensing by DNA-PK have been described [29,30]. Another cytoplasmic function of DNA-PK is its participation in translation regulation, in particular Ku has been found to bind p53 mRNA and this results in repression of p53 protein synthesis [31]. Of note, the need for phosphorylating activity of DNA-PK for this process has not been established. 

In addition, there is a number of other cellular processes involving the components of the DNA-PK complex, however their role cannot be explained by the mechanical ability of DNA-PK to bind the ends of DNA. DNA-PK is shown to be implicated in the regulation of mitosis, telomere maintenance, hypoxic response, metabolism, and transcription regulation. Some of these issues are discussed in detail in other reviews [2,3,6,32]. The involvement of DNA-PK in the regulation of transcription of cellular genes and HIV-1 is discussed in detail below in Section 3 and Section 4, respectively.

## 3. Phosphorylation Targets of DNA-PK 

As mentioned above, DNA-PK kinase activity is important for various cellular processes. Although the components of NHEJ pathway exposed to phosphorylation have been identified [16,33,34,35,36,37,38,39,40,41,42,43] and are discussed below, the detailed role of DNA-PK catalytic activity in DSB repair and other processes is not yet completely understood, and our knowledge of phosphoproteome alterations upon DNA-PK activation is not complete.

We found and analyzed DNA-PK targets described in 63 articles, in which the relation between DNA-PK activation and phosphorylation of a human protein is revealed. These studies describe 67 unique human proteins phosphorylated by DNA-PK (Table 1). For 22 of them, phosphorylation has been described in vitro, for 25 of them only in vivo, and for 20 targets both in vitro and in vivo. 

The kinase assay using [γ-^32^P]-ATP was the most frequent method to detect phosphorylation in vitro. In this case, a putative target protein was incubated with DNA-PK complex components under appropriate conditions including the activator of DNA-PKcs. Phosphorylation events were detected by ^32^P-incorporation into a band in SDS-PAGE that corresponds to the target protein (Table 1, ^32^P-incorporation). In the case of in vivo targets identification, phosphorylation of proteins was analyzed using Pro-Q Diamond, which is a specific dye for phosphoproteins, followed by mass-spectrometry (Table 1, ProQ-Diamond staining + MS). In this case, the protein that is differentially phosphorylated in the experiment vs. control can be identified, but the identification of phosphorylated peptides needs additional research, which is why phosphorylated amino acid residues have not been identified for some of the targets in Table 1. The site-directed mutagenesis was also used to confirm identified amino acid residues as phosphorylation sites (Table 1, mutagenesis). When it was possible to obtain antibodies against phosphorylated forms of targets, they were used to investigate the details of their phosphorylation (Table 1, WB (Western blot) and IF (immunofluorescence)). In rarer cases, protein phosphorylation was detected by changes in the mobility of the target protein in SDS-PAGE, which is applicable both for in vitro and in vivo studies (Table 1, changes in gel mobility of phosphorylated forms). 

DNA end mimicking molecules such as Dbait32H (in vitro and in vivo studies), sheared genomic DNA (in vitro activation), etc. were commonly used as DNA-PK activators: upon their introduction, phosphorylation of the targets was observed (Table 1). Besides, DNA-PK targets were phosphorylated upon exposure to classical DNA-damaging agents: ionizing radiation (IR), UV, bleomycin, etoposide, camptothecin, doxorubicin, zeocin, calicheamicin γ1, and 4-nitroquinoline-1-oxide (4NQO) (Table 1). In some studies, DNA-PKcs activation and the subsequent phosphorylation of the targets were observed in response to non-canonical stimuli: C1D protein, heterogeneous ribonucleoprotein particle (hnRNP), and telomerase RNA component (hTR) (Table 1).

Phosphorylation sites were identified for 29 of the 67 described proteins (Table 1), most of them belonging to SQ or TQ motifs—sequences found in proteins phosphorylated by the members of PIKKs family: ATM, ATR, and DNA-PKcs [1]. Three (T2624, S3205, and S4026) out of nine DNA-PKcs autophosphorylation sites are not located in SQ or TQ sites [37]. The DNA-PKcs phosphorylation sites on Ku70, Ku86, and XRCC4 are also located in sites other than this consensus [35,40]. 

DNA-PK-dependent phosphorylation of the proteins with the determined phosphorylation sites had various effects on their functionality. Thus, phosphorylation of well-known DNA-repair proteins NHEJ1 [40] and XRCC4 [40,43] was dispensable for DNA repair in vivo. In contrast, phosphorylation of such proteins as ATM [19], DCLRE1C [37], GOLPH3 [51], H2AFX [90], HSP90AA1 [63], NR4A2 [70], POU2F1 [75], PNKP [72], RPA2 [77], and WRN [39] was important for the DNA-repair regulation. Phosphorylation of ATM leads to its inhibition, which may explain how the cell makes its choice between homologous recombination, stimulated by ATM, and NHEJ, given that DNA-PKcs and ATM are both simultaneously recruited and activated at the same DSB ends [19]. C-terminal phosphorylation of Artemis increases its endonucleolytic activity that may affect DNA-end processing in NHEJ-pathway [37]. T7-phosphorylated Hsp90α is accumulated at the site of DNA damage, where it appears to be important for maintaining phosphorylated histone H2AX [63]. Mutations in WRN that impair DNA-PK-dependent phosphorylation change the kinetics of WRN dissociation from DSBs and decrease the efficiency of DSB repair [39]. 

A well-known transcription factor POU2F1 (Oct-1) was phosphorylated by DNA-PK under IR or zeocin treatment, and this event regulated Oct-1 dependent transcription, leading to an increased cell survival after DNA damage [75]. The same effects on the cell survival were observed in the case of DNA-PK-dependent phosphorylation of GOLPH3, that regulates Golgi dispersal [51]. Interestingly, the phosphorylation of another transcription factor NR4A2 promotes DSB repair, but NR4A2 transcriptional activity is entirely dispensable in this function. Instead, NR4A2 functions directly at DNA repair sites [70]. Human polynucleotide kinase/phosphatase (PNKP) has the dual function as 5’-DNA kinase and 3’-DNA phosphatase and plays a role in NHEJ and other DNA repair processes. The phosphorylation of PNKP on S114 and S126 in DNA-PK and ATM-dependent manner was demonstrated in vitro, and was confirmed on S126 in vivo. This phosphorylation promotes the retention of PNKP at sites of DNA damage, and may regulate its catalytic activity near DSB sites that helps to successfully complete the DNA repair process [72]. The phosphorylation events described above are important for the successful NHEJ. Moreover, the phosphorylation of RPA32 at S4/S8 by DNA-PKcs is essential for cell survival under replicative stress [77]. Even this shows that the role of the kinase activity of DNA-PK goes beyond the NHEJ-pathway. 

In several reports, protein translocation after phosphorylation was detected, and this may be important for the cellular response to DNA-damaging agents [16,44,50]. For example, a cell treatment with antibiotic calicheamicin γ1, which causes DNA double-strand breaks, led to cytoplasmic accumulation of FUS, which depended on phosphorylation of its N-terminus by DNA-PK [50]. Besides, regulation of cell mobility and adhesion was observed in the case of DNA-PK-dependent phosphorylation of VIM under Dbait32H treatment [16].

We performed a functional enrichment analysis of DNA-PK targets to elucidate the cellular processes, in which DNA-PK targets are involved. Annotation of 67 genes was performed using the DAVID online analysis tool v 6.8 [91]. Genes were divided into 17 functional clusters with enrichment score higher than 1.7 (Table S1). Most of them were enriched with following terms: DNA-repair (36 targets), nucleotide binding (26 targets), DNA-binding (26 targets), transcription regulation (23 targets), regulation of cellular response to heat (8 targets), RNA processing and splicing (8 targets), PI3K-Akt signaling pathway (8 targets), telomere maintenance (7 targets), cell cycle (7 targets), DNA-unwinding (7 targets), and cytoskeleton (6 targets). 

**Figure 2:**
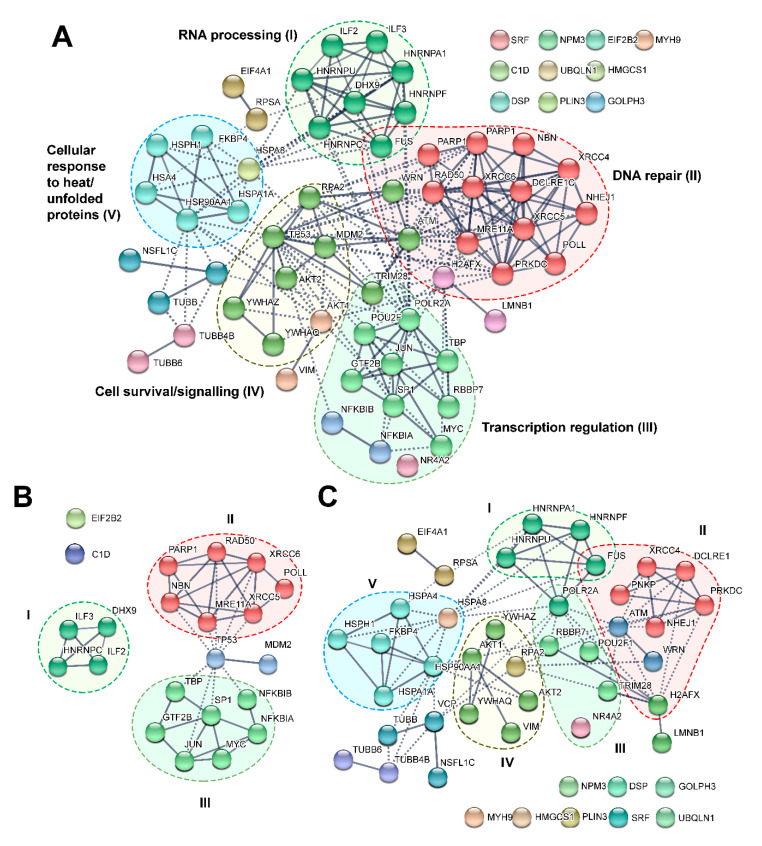
Protein–protein interactions network of DNA-PKcs targets described in Table 1 (**A**). PPI networks of DNA-PKcs targets identified only in in vitro experiments (**B**) and in in vivo or both in vitro and in vivo experiments (**C**). The most connected nodes are the same color and are grouped in a cluster using Markov Cluster Algorithm (MCL) with inflation parameter 3.1.

To determine protein–protein associations of DNA-PK targets listed in Table 1, we used STRING database *v.* 11.0 [92] and revealed a network of directly interacting proteins. We excluded the “*text mining*” option from the active interaction sources because of the low level of reliability of this type of data, and also because our protein targets were retrieved by the articles’ analysis. It turned out that, among 67 proteins, 56 were directly interconnected (Figure 2A). The resulting network had meaningfully more interactions than expected (218 vs. 64 expected edges) with protein–protein interactions (PPI) enrichment *p*-value < 10^−16^. Markov Cluster Algorithm (MCL) analysis revealed five main clusters of the most interconnected nodes. 

The proteins in these clusters could be divided according to their functions into DNA repair related proteins, transcription regulation, cell response to heat or unfolded proteins, RNA processing, and cell survival/signaling factors (Figure 2A). Of note, the mathematical approach to protein clusterization in the interaction network (MCL analysis) led to practically the same results as the functional classification.

Taking into account that our target list (Table 1) contained some proteins for which DNA-PK-dependent phosphorylation has been confirmed only in vitro, we analyzed “in vitro” and “in vivo” target groups separately, 22 and 45 targets, respectively. The second group contained both in vivo and in vitro/in vivo confirmed targets. Analysis of the “in vitro” group showed significant PPI enrichment: *p*-value < 10^−16^, 43 vs. 7 expected edges. This group can be divided into three clusters containing at least four proteins. The clusters contain proteins with the following functions: DNA-repair, transcription regulation, and RNA-processing (Figure 2B). For the “in vivo” group (PPI enrichment *p*-value < 2.22 × 10^−16^, 80 vs. 27 expected edges), five clusters were identified. According to their functions, these proteins could be classified as DNA-repair, RNA processing, cell response to heat or unfolded proteins, and cell survival/signaling factors (Figure 2C). The full list of the enriched biological processes and molecular functions (GO) is presented in Table S2.

The analysis of Table 1 and Figure 2 demonstrates that most of the research of DNA-PK targets was focused on proteins anyway involved in DNA repair processes. This is not surprising, given that the role of DNA-PK in DNA repair is studied best. Furthermore, it was a priori assumed that DNA-PK is activated only upon binding to DNA ends forming as a result of a double-strand break. However, DNA-PK may also be activated in the absence of DNA ends [5,9,46]. For instance, hTR dependent phosphorylation of HNRNPA1 by DNA-PK is essential for telomere maintenance [5]. Although these studies are not numerous, the variety of systems with confirmed DNA-independent activation of DNA-PK lends credibility to these data. All these data additionally demonstrate that DNA-PK may regulate a broad spectrum of cellular processes. In particular, the ability of DNA-PK to become activated upon binding to RNA may explain the presence of a substantial number of target proteins involved in RNA processing in the “in vitro”, as well as in the “in vivo” group. 

As shown in Figure 2B, among DNA-PK phosphorylation targets identified in vitro, there is a significant group consisting of proteins involved in transcription regulation, such as TBP, JUN, MYC, etc. Transcription regulation factors can also be found in the “in vivo” group (POU2F1, TRIM28, POLR2A, NR4A2, and RBBP7). Such targets seem quite plausible, given that the association between activation of DDR kinases (ATM and DNA-PKcs) and transcriptional regulation has already been demonstrated. On the one hand, transcription from certain promoters is initiated through the introduction of DSB by topoisomerase IIβ, and DNA-PK is involved in the repair of such breaks [93]. It was also shown that expression regulation of some stimulus-inducible and developmental genes can occur by RNA polymerase II (RNAP II) pausing and pause release [94]. Typically, RNAP II pauses at around +30–100 relative to the transcriptional start site until activating cellular signals induce elongation [95]. Interestingly, the DNA-repair factor TRIM28 is a main player that maintain RNAP II paused state. To release RNAP II, topoisomerase II should introduce the DSB at the regulated genes. This event activates DDR kinases ATM and DNA-PKcs, which phosphorylate protein targets including H2AX and TRIM28. The phosphorylated TRIM28 is no longer able to retain RNAP II near the transcriptional start, which results in processive elongation of RNAP II and effective synthesis of RNA [13,87,94]. Interestingly, these transcription-induced, topoisomerase II-mediated DSBs can also be exploited therapeutically and propose that, in hormone-dependent tumors such as breast and prostate cancers, a hormone-cycling therapy, in combination with topoisomerase II poisons or inhibitors of the DNA repair component DNA-PKcs, could overwhelm cancer cells with transcription-associated DSBs [96]. On the other hand, the occurrence of DSBs in a human gene transcribed by RNAP II is known to lead to inhibition of transcription elongation and reinitiation. Upon inhibition of DNA-PK (or ATM) activity, RNAP II bypasses the break and continues transcription elongation, suggesting that it is not the break per se that inhibits the processivity of RNAP II, but the activity of the kinases [97,98]. However, it is known that DNA-PK-dependent phosphorylation of such NF-κB inhibitors as IκBα (NFKBIA) and IκBβ (NFKBIB) favors the association of NF-κB with DNA [68]. It has been shown in other studies that NF-κB is activated as part of the DNA damage response and is thought to orchestrate a cell survival pathway, which, together with the activation of cell cycle checkpoints and DNA repair, allows the cell in cases of limited damage to restore a normal life cycle [99]. DNA-PK likely utilizes several mechanisms to regulate transcription and is capable of exerting both positive and negative effects.

## 4. DNA-PK Targets in HIV Replication

HIV-1 replication cycle is conventionally divided into early and late replication stages. The early stages are aimed at the formation of proviral DNA, i.e., that of the copy of the viral genome integrated into the genome of the infected cell. They include viral attachment and entry, reverse transcription, nuclear import, integration, and post-integrational DNA repair (PIR) [11,100]. The late stages include virion component biosynthesis and assembly of new viral particles [100]. The dependence of the HIV-1 replication on the DNA-PK-complex components has been previously shown by different groups [10,101,102,103,104,105,106,107]. We previously showed that the phosphorylating activity of DNA-PKcs was necessary for HIV-1 PIR [10]. To search for possible downstream proteins involved in this process, we analyzed the data on the role of DNA-PK targets in Table 1 in HIV-1 replication.

For 27 proteins out of 67 represented in Table 1, information on their involvement in HIV-1 replication cycle is available. Thus, 13 proteins favor HIV-1 replication, 6 proteins impair it, data for 3 proteins are controversial, and 5 proteins have opposite effects on HIV-1 replication, depending on the life cycle stage (Table 2). Table 2 also provides information on HIV-1 life cycle stages influenced by these factors.

It has been suggested that certain factors are involved in post-integrational DNA repair or integration (Table 2). Unfortunately, the discrimination of their effects on each of these two stages was not possible up until recently, since there were no methods allowing the quantitative assessment of post-integrational DNA repair. That is why the effects of cellular proteins on the repair were studied only by indirect methods, for instance, based on the decrease of integrated HIV-1 DNA levels 48 hpi vs. 24 hpi [108]. Only three proteins, DHX9, EIF4A1, and p53 (TP53), have been shown to participate in reverse transcription, the latter being also involved in other stages of viral life cycle (Table 2). However, the significance of phosphorylation of these proteins for their functionality in HIV-1 replication has not yet been demonstrated.

Here, we dwell on DNA-PK phosphorylation targets involved in post-integrational DNA repair, starting with DNA-PKcs (PRKDC) itself, as well as Ku70 (XRCC6) and Ku80 (XRCC5), which form DNA-PK complex together. Their beneficial effect on HIV-1 replication was shown in numerous studies [101,102,103,104,105,106]. It was speculated that DNA-PK complex is involved in PIR and our method of the quantitative assessment of PIR efficiency [12] allowed directly proving the involvement of this complex in this process [12].

Based on indirect data, kinase ATM was supposed to be involved in PIR [108]. Thus, the presence of small-molecule ATM inhibitor Ku55933 was shown to have no effect on the level of integrated viral DNA at 24 hpi; however, at 48 hpi, the levels of integrated DNA in inhibitor-treated cells drastically decreased, but remained practically unchanged in the control cell line [108]. The authors attributed this effect to the impossibility of post-integrational DNA repair with ATM being inhibited. The role of MRN complex components MRE11 and Nibrin (NBN), the main factors recruiting ATM to DNA damage sites [109], in the replicative cycle is controversial [106,110]. Thus, Smith et al. showed that the decreased levels of MRN complex components decreases the efficiency of transduction with an HIV-1-based vector [110], whereas, in the study by Sakurai et al., the transduction of cells with defective components of this complex was similar to that in control cell lines [106]. However, integration site sequencing revealed the higher mutation rate in integration sites in the absence of NBN and MRE11, which supports the dysfunctional repair in these cells [106]. It may indirectly support these proteins being involved in the PIR of proviral DNA.

PARP-1 is another DNA-PK target, the role of which in HIV-1 replication has been actively studied, but, to date, the results are rather controversial [153]. Some studies [130,131,132,133] show that PARP-1 plays an important role in HIV-1 integration. At the same time, the importance of PARP-1 for retroviral replication is doubted in other studies [134,135,136]. More recent research shows that PARP-1 is involved in HIV-1 replication in the step of repression of transcription from the provirus, but it does not affect the integration level [138,154]. Nevertheless, various studies describe the beneficial effect of PARP-1 on the transcription from the HIV-1 promoter [130,137,155], as well as its negative effect [138,154,156]. However, the data on the negative effect of PARP-1 on transcription were obtained in a chicken B lymphoblastoid cell system, whereas the beneficial effect was observed in human cells (HeLa, J111 and human primary monocyte-derived macrophages), which is a more relevant model for studying HIV-1.

Histone H2AX (H2AFX gene), another DNA-PK target, is phosphorylated upon retroviral integration; however, this event is not likely to be necessary for efficient replication [119], but can be used as a marker for PIR research.

Most proteins in Table 2 regulate transcription from the LTR promoter, i.e., one of late stages of the replication cycle. HIV-1 genes are transcribed by RNAP II (POLR2A is a part of this complex) from a viral promoter in the 5′LTR (long terminal repeat). Besides the promoter, the 5′LTR contains a modulator sequence and an enhancer, interacting with a number of transcription factors regulating the transcription of HIV genes. The provirus may be latent or actively transcribed. The latent state may be characterized by the absence of transcription from the LTR promoter or by synthesis of short abortive fragments of 60–80 nucleotides, forming a stable RNA hairpin TAR (trans-activation response element) [157]. After TAR RNA synthesis, RNAP II stops, being bound to factors repressing transcription elongation, namely NELF (negative elongation factor) and DSIF (5,6-dichloro-1-β-D-ribofuranosylbenzimidazole sensitivity-inducing factor) [158]. For the transition into the state of active transcription, the C-terminal domain of RNAP II needs to be hyperphosphorylated on S2. The key participant of this process is P-TEFb complex (positive transcription elongation factor), recruited to HIV-1 promoter by direct interaction with Tat viral protein bound to TAR RNA [159]. However, there is increasing evidence of DNA-PK involvement in this process.

In particular, it is known that DNA-PK may phosphorylate the C-terminal domain of RNAP II [74], and, along with the Ku70/Ku80 heterodimer, DNA-PKcs may be precipitated from human cells together with the RNAP II holoenzyme [160]. It has also been shown that the Ku70/Ku80 heterodimer may interact with hairpin RNA involved in transcription performed by RNAP II, HIV-1 TAR PHK and 7SK PHK [159,161,162], whereas the catalytic subunit of DNA-PKcs forms a complex with HIV-1 Tat [163] and may phosphorylate it [164]. These various data indicate the possible involvement of the DNA-PK DNA repair complex in the regulation of transcription from the HIV-1 promoter. Of note, S. Tyagi et al. demonstrated the parallel distribution of DNA-PKcs with RNAP II along the HIV-1 provirus before and after transcription activation with tumor necrosis factor alpha. Interestingly, when the provirus changes its state from latent to active, the levels of both DNA-PKcs and RNAP II associated with the HIV-1 promoter increase dramatically [164]. Finally, it has recently been shown that, in different cell lines, DNA-PK increases the phosphorylation of RNAP II C-terminal domain at S5 and S2 by directly catalyzing phosphorylation and by augmenting the recruitment of P-TEFb at HIV LTR. Thus, the DNA-PK-dependent phosphorylation of RNAP II likely plays an important role in both transcription initiation and elongation [14]. Considering the role of Top II and DNA-PKcs in pause release of RNAP II through the DSB formation in cellular stimuli induced genes, one may assume that LTR-driven transcription may also be regulated in the same manner. Although this idea is more speculative and needs further testing, TRIM28, which is main maintainer of paused RNAP II, has been demonstrated to participate in HIV latency by SUMOylating CDK9 and inhibiting P-TEFb [149]. Moreover, DNA-PK utilizes the mechanism of the TRIM28 recruitment at LTR and its phosphorylation to release paused RNAP II, thus influencing different steps of transcription from the LTR promoter [14]. 

LTR is an intricate regulatory sequence of the HIV-1 genome. It contains binding sites of such transcription regulators as AP-1 complex (JUN), c-MYB, NFAT, GR (NR3C1), USF1, ETS1, LEF1, CEBP, NF-κB, SP1, TBP, UBP1, UBP2, CTF/NF1, and IRF [141]. Some of these factors, such as JUN, SP1, and TBP, may be phosphorylated by DNA-PK in response to various stimuli (Table 1). The phosphorylated TBP and TFIIB synergistically stimulate RNAP II basal transcription from adenovirus major late promoter, which means that DNA-PK can positively regulate the RNAP II basal transcription by phosphorylating TBP and TFIIB [52]. It has been shown that Sp1 forms a tight protein–protein complex with viral Tat, and both proteins in this complex are phosphorylated by DNA-PK. Importantly, it is the phosphorylation status of Sp1 and not the levels of LTR-promoter-bound Sp1 that affects HIV-1 transcription [165]. It was previously shown that DNA-PK-dependent phosphorylation of NF-κB inhibitors IκBα (NFKBIA) and IκBβ (NFKBIB) promotes the association of NF-κB with DNA [68]. Therefore, DNA-PK associated with the LTR promoter may favor LTR-dependent HIV-1 transcription by promoting the binding of NF-κB with LTR.

## 5. Conclusions

In this work, we tried to systematize the literature data on proteins phosphorylated by DNA-PK, having two goals: to understand in which cellular processes the phosphorylating activity of this kinase may be important and to find potential participants of PIR among DNA-PK targets. The results of our work undoubtedly show the variety of DNA-PK’s functions, not limited by participation in DNA double-strand break repair by NHEJ pathway. In the case of HIV-1, it is involved in at least two stages of the replication cycle of the virus: post-integrational DNA repair and regulation of transcription from the LTR promoter. Unfortunately, the analysis of the targets of DNA-PK related to HIV-1 replication cycle (Table 2) does not clearly reveal the participants of the PIR process exposed to phosphorylation. Assuming PIR may involve proteins from other repair processes, the most likely candidates are ATM, Artemis (DCLRE1C), MRE11, NBN, and XRCC4. However, further research is clearly needed to exactly define the events of HIV-1 PIR occurring upon DNA-PKcs activation.

## Figures and Tables

**Table 1:** DNA-PK targets and effects of their phosphorylation.

Gene Name	Protein Name	Type of Experiment	DNA-PKcs Activation Method	Phosphorylation Event Validation	Identified Phosphorylation Sites	Effect of Phosphorylation (In Vivo)	Ref.
Akt1; Akt2; Akt3	RAC-alpha serine/threonine-protein kinase	in vitro/in vivo	CpG ODN, UVB	Phosphorylated Akts activity; WB; ^32^P-incorporation	AKT1: T308, S473; AKT2: T309	Cell survival after UVB treatment, Akt translocation to nucleus after CpG ODN treatment	[44,45]
ATM	Serine-protein kinase ATM	in vitro/in vivo	bleomycin	WB; ^32^P-incorporation; mutagenesis	S85/T86, T372/T373 and S1985/T1987/T1988	Negative regulation of ATM	[19]
C1D	Nuclear nucleic acid-binding protein C1D	in vitro	C1D, dsDNA	^32^P-incorporation			[46]
DCLRE1C	Protein Artemis	in vitro/in vivo	dsDNA, bleomycin	MS; changes in gel mobility of phosphorylated forms; ^32^P-incorporation; WB	S385, T410, S417, S503, S509, S516, S518, S572, S589, T601, S645, T676, S679, S688, T692	Increase Artemis association with chromatin	[37,47,48]
DHX9	ATP-dependent RNA helicase A	in vitro	poly(rG)	^32^P-incorporation			[9]
DSP	Desmoplakin	in vivo	Dbait32H	ProQ-Diamond staining + MS			[16]
EIF2B2	Translation initiation factor eIF-2B subunit beta	in vitro	dsDNA	^32^P-incorporation			[49]
EIF4A1	Eukaryotic initiation factor 4A-I	in vivo	Dbait32H	ProQ-Diamond staining + MS			[16]
FKBP4	Peptidyl-prolyl cis-trans isomerase FKBP4	in vivo	Dbait32H	ProQ-Diamond staining + MS			[16]
FUS	RNA-binding protein FUS	in vitro/in vivo	Calicheamicin γ1, Dbait32H	WB; changes in gel mobility of phosphorylated forms; mutagenesis	S/T-Q located in N-terminal region of FUS (1–165 aa)	Translocation to cytoplasm	[50]
GOLPH3	Golgi phosphoprotein 3	in vitro/in vivo	Camptothecin, doxorubicin, IR	MS; ^32^P-incorporation	T143	Cell survival following DNA damage	[51]
GTF2B	Transcription initiation factor IIB	in vitro	dsDNA	^32^P-incorporation			[52]
H2AFX	Histone H2AX	in vitro/in vivo	dsDNA, IR, Dbait32H	WB; IF; changes in gel mobility of phosphorylated form	S139	Assembly of DNA repair proteins at the DNA-damage sites	[16,53,54,55,56,57,58]
HMGCS1	Hydroxymethylglutaryl-CoA synthase, cytoplasmic	in vivo	Dbait32H	ProQ-Diamond + MS			[16]
HNRNPA1	Heterogeneous nuclear ribonucleoprotein A1	in vitro/in vivo	dsDNA, hTR, hnRNP	^32^P-incorporation; WB; mutagenesis	S95, S192	Essential for capping of the newly replicated telomeres and prevention of telomeric aberrations	[5,9,59]
HNRNPC	Heterogeneous nuclear ribo-nucleoproteins C1/C2	in vitro	hnRNP	^32^P-incorporation			[9]
HNRNPF	Heterogeneous nuclear ribo-nucleoprotein F	in vivo	Dbait32H	ProQ-Diamond + MS			[16]
HNRNPU	Heterogeneous nuclear ribo-nucleoprotein U	in vitro/in vivo	dsDNA, etoposide, Calicheamicin γ1	MS; WB; changes in gel mobility of phosphorylated form	S59		[60,61]
HSP90AA1	Heat shock protein HSP 90-alpha	in vitro/in vivo	dsDNA; Dbait32H; IR	ProQ-Diamond + MS; ^32^P-incorporation; WB	T5, T7	pThr7-HSP90α accumulates at repair foci, that is necessary for maintenance of γ-H2AX Foci and efficient DNA repair	[16,62,63,64]
HSPA1A	Heat shock 70 kDa protein 1A	in vivo	Dbait32H	ProQ-Diamond + MS			[16]
HSPA4	Heat shock 70 kDa protein 4	in vivo	Dbait32H	ProQ-Diamond + MS			[16]
HSPA8	Heat shock cognate 71 kDa protein	in vivo	Dbait32H	ProQ-Diamond + MS			[16]
HSPH1	Heat shock protein 105 kDa	in vivo	Dbait32H	ProQ-Diamond + MS			[16]
ILF2	Interleukin enhancer-binding factor 2	in vitro	dsDNA	^32^P-incorporation			[49]
ILF3	Interleukin enhancer-binding factor 3	in vitro	dsDNA	^32^P-incorporation			[49]
JUN	Transcription factor AP-1	in vitro	dsDNA	^32^P-incorporation; mutagenesis	S249		[65]
LMNB1	Lamin-B1	in vivo	Dbait32H	ProQ-Diamond + MS			[16]
MDM2	E3 ubiquitin-protein ligase Mdm2	in vitro	dsDNA	^32^P-incorporation; mutagenesis	S17	Mdm-2 Phosphorylation by DNA-PK Prevents Interaction with p53	[66]
MRE11	Double-strand break repair protein MRE11	in vitro	dsDNA	^32^P-incorporation			[19]
MYC	Myc proto-oncogene protein	in vitro	dsDNA	^32^P-incorporation			[67]
MYH9	Myosin-9	in vivo	Dbait32H	ProQ-Diamond + MS			[16]
NBN	Nibrin	in vitro	dsDNA	^32^P-incorporation			[19]
NFKBIA	NF-kappa-B inhibitor alpha	in vitro		^32^P-incorporation; MS	S36, T273		[68]
NFKBIB	NF-kappa-B inhibitor beta	in vitro		^32^P-incorporation			[68]
NHEJ1	Non-homologous end-joining factor 1 (XLF)	in vitro/in vivo	dsDNA, IR	MS; WB; ^32^P-incorporation	S245	Dispensable for DSB repair	[69]
NPM3	Nucleoplasmin-3	in vivo	Dbait32H	ProQ-Diamond + MS			[16]
NR4A2	Nuclear receptor subfamily 4 group A member 3	in vitro/in vivo	dsDNA, IR	WB; MS; IF; mutagenesis	S337	Promotes DSB repair	[70]
NSFL1C	NSFL1 cofactor p47	in vivo	Dbait32H	ProQ-Diamond + MS			[16]
PARP1	Poly-(ADP-ribose) polymerase 1	in vitro	dsDNA	^32^P-incorporation			[71]
PLIN3	Perilipin-3	in vivo	Dbait32H	ProQ-Diamond + MS			[16]
PNKP	Bifunctional polynucleotide phosphatase/kinase	in vitro/in vivo	IR	WB; MS; ^32^P-incorporation; mutagenesis	S114, S126	Regulates DSB repair	[72]
POLL	DNA polymerase lambda, involved in BER, NHEJ and HR	in vitro	dsDNA	WB; ^32^P-incorporation; mutagenesis	T204		[73]
POLR2A	DNA-directed RNA polymerase II subunit RPB1	in vitro/in vivo	dsDNA; unknown transcriptiona signal	WB; ^32^P-incorporation	Heptapeptide repeats of CTD; S2; S5	Increase transcription efficiency	[14,74]
POU2F1	POU domain, class 2, transcription factor 1 (octamer transcription factor 1, Oct-1)	in vitro/in vivo	dsDNA, IR, zeocin	^32^P-incorporation		Stabilizes Oct-1, decreases Oct-1 dependent transcription	[75]
PRKDC	DNA-dependent protein kinase catalytic subunit	in vivo	Dbait32H	WB; IF; MS; ^32^P-incorporation; mutagenesis	S2056, T2609, S2612, T2620, S2624, T2638, T2647; S3205; S3821; S4046; T4102		[16,33,36,37]
RAD50	DNA repair protein RAD50	in vitro	dsDNA	^32^P-incorporation			[19]
RBBP7	Histone-binding protein RBBP7	in vivo	Dbait32H	ProQ-Diamond + MS			[16]
RPA2	Replication protein A 32 kDa subunit	in vitro/in vivo	Camptothecin, UV, 4NQO, Etoposide	WB; mutagenesis; ^32^P-incorporation; changes in gel mobility of phosphorylated forms	S4, S8, T21	Regulates fork restart, new origin firing, HR, mitotic catastrophe, and cell survival in response to replication stress. RPA2 hyperphosphorylation by DNA-PK in response to DSBs blocks unscheduled homologous recombination and delays mitotic entry.	[76,77,78,79]
RPSA	40S ribosomal protein SA	in vivo	Dbait32H	ProQ-Diamond + MS			[16]
SP1	Transcription factor Sp1	in vitro	dsDNA	^32^P-incorporation			[80]
SRF	Serum response factor	in vitro/in vivo	IR	^32^P-incorporation; two-dimensional separation of phosphopeptides on thin-layer cellulose plates	S435, S446		[81]
TBP	TATA-box-binding protein	in vitro	dsDNA	^32^P-incorporation			[52]
TP53	Cellular tumor antigen p53	in vitro	dsDNA	WB; ^32^P-incorporation; SPR	S6, S15, S37, S46, S166		[82,83,84,85]
TRIM28	Transcription intermediary factor 1-beta	in vivo	IR; Heat-shock induced gene transcription	WB	S824	TRIM28 phosphorylation induces chromatin changes in response to DNA breaks.	[13,86,87]
						TRIM28 stabilizes Pol II pausing, and its release depends on the S824 phosphorylation.	
TUBB	Tubulin beta chain	in vivo	Dbait32H	ProQ-Diamond + MS			[16]
TUBB2C	Tubulin beta-4B chain	in vivo	Dbait32H	ProQ-Diamond + MS			[16]
TUBB6	Tubulin beta-6 chain	in vivo	Dbait32H	ProQ-Diamond + MS			[16]
UBQLN1	Ubiquilin-1	in vivo	Dbait32H	ProQ-Diamond + MS			[16]
VCP	Transitional endoplasmic reticulum ATPase	in vivo	Dbait32H	ProQ-Diamond + MS			[16]
VIM	Vimentin	in vitro/in vivo	Dbait32H	ProQ-Diamond + MS; WB; ^32^P-incorporation	S459	Regulates cell migration and adhesion	[16]
WRN	Werner syndrome ATP-dependent helicase	in vitro/in vivo	dsDNA; bleomycin; 4NQO	WB; MS; ^32^P-incorporation; mutagenesis	S440, S467	Inhibits both the helicase and exonuclease activities of WRN. Phosphorylation of S440 and S467 are important for relocalization of WRN to nucleoli, and that it is required for efficient DSB repair.	[38,39,88]
XRCC4	DNA repair protein XRCC4	in vitro/in vivo	dsDNA, IR	MS; ^32^P-incorporation; mutagenesis	S260, S318, S320	Not essential for DSB repair	[34,40,41,42,43]
XRCC5	XRCC5 X-ray repair cross-complementing protein 5 (Ku80)	in vitro	dsDNA	WB; MS; ^32^P-incorporation; amino acid sequencing	S577, S580, T715		[35,89]
XRCC6	XRCC6 X-ray repair cross-complementing protein 6 (Ku70)	in vitro	dsDNA	WB; MS; ^32^P-incorporation; amino acid sequencing	S6		[35,89]
YWHAQ	14-3-3 protein theta	in vivo	Dbait32H	ProQ-Diamond + MS			[16]
YWHAZ	14-3-3 protein zeta/delta	in vivo	Dbait32H	ProQ-Diamond + MS			[16]

**Table 2:** Effects of DNA-PKcs targets on HIV-1 replication.

Gene Name (Common Protein Name)	Role in HIV Life Cycle	Comments/Life Cycle Step	Publications
AKT	Positive	Cell survival during HIV infection	[111,112]
ATM	Positive	Post-integrational repair (indirect evidence)	[101,106,108,113]
DCLRE1C (Artemis)	Positive	No data	[114]
DHX9	Positive	Reverse transcription	[115,116]
EIF4A1	Positive	Reverse transcription	[117]
FUS	Negative	LTR-dependent transcription	[118]
H2AFX (H2AX)	Dispensable	H2AFX is phosphorylated during integration, but not essential for HIV replication	[119]
HNRNPA1	Complex	HIV transcription, viral mRNA splicing, mRNA transport	[120,121]
HSP90AA1	Positive	Transcription, capsid core stability	[122]
JUN	Complex	Transcription. c-Jun enhances Tat-mediated LTR transcription but suppresses basal LTR transcription without Tat	[123]
MDM2	Complex	1. Positive regulator of early replicative stages in macrophages by inhibition of p53 activity;	[124,125,126]
		2. Negative regulation of Vif stability, removes its counteracting effect on the APOBEC3G restriction factor;	
		3. Positive regulation of Tat activity	
MRE11	Controversial	Integration, pre-integration steps, post-integrational DNA repair (indirect and controversial evidences)	[106,110]
MYC	Complex	1. Positive regulation of cDNA nuclear transport;	[127,128]
		2. c-Myc and Sp1 contribute to proviral latency. Negative regulation of transcription from LTR promoter	
NBN (Nibrin)	Controversial	Integration, pre-integration steps, post-integrational DNA repair (indirect and controversial evidences)	[106,110]
NFKBIA (IκBα)	Negative	IκBα but not IκBβ suppress latent-active transcription transition	[129]
PARP1	Controversial	1. Early replicative steps (integration and/or post-integrational DNA-repair (indirect evidence)	[130,131,132,133,134,135,136,137,138]
		2. LTR-dependent transcription	
POU2F1 (Oct-1)	Negative	Repress LTR-mediated transcription	[139]
RBBP7	Negative	LTR-mediated transcription	[140]
SP1	Positive	c-Myc and Sp1 contribute to proviral latency	[141]
TBP	Positive	LTR-mediated transcription	[142,143]
TP53 (p53)	Negative	1. Reverse transcription	[144,145,146,147,148]
		2. LTR-mediated transcription	
		3. Cell survival during HIV-infection	
TRIM28	Negative	Promotes HIV-1 Latency. DNA-PKcs dependent phosphorylation reactivates LTR mediated transcription	[14,149]
VIM	Positive	No data	[150]
WRN	Positive	LTR-mediated transcription	[151,152]
XRCC4	Positive	early replicative stages	[103]
XRCC5 (Ku80)	Complex	1. LTR-mediated transcription	[10,101,102,103,104,105,106,107]
		2. Integration, post-integrational DNA repair (direct evidence)	
XRCC6 (Ku70)	Positive	Post-integrational DNA repair (direct evidence)	[10]

## Supplementary Materials

The following are available online at https://www.mdpi.com/2073-4409/9/8/1907/s1, Table S1: DAVID functional annotation of DNA-PKcs targets, Table S2: Enriched biological processes and molecular functions in DNA-PKcs targets list.
